# Baicalin improves podocyte injury in rats with diabetic nephropathy by inhibiting PI3K/Akt/mTOR signaling pathway

**DOI:** 10.1515/med-2021-0335

**Published:** 2021-09-02

**Authors:** Yi Ou, Wenjuan Zhang, Shaopeng Chen, Haihua Deng

**Affiliations:** Department of Endocrinology, Shenzhen Fuyong People’s Hospital, Shenzhen, Guangdong 518103, China; Department of Public Health and Preventive Medicine, School of Basic Medical Sciences, Jinan University, Guangzhou, Guangdong 510632, China; Department of Biomedicine, School of Medicine, Shenzhen University, Shenzhen, Guangdong 518061, China; Department of Neurology, Shenzhen Fuyong People’s Hospital, Shenzhen, Guangdong 518103, China

**Keywords:** diabetic nephropathy, podocytes, baicalin, PI3K/Akt/mTOR signaling pathway

## Abstract

**Objective:**

To investigate the effect of baicalin on diabetic nephropathy (DN) rats and podocytes and its mechanism.

**Methods:**

The rat models with DN were established by high-fat and high-sugar diet and intraperitoneal injection of streptozotocin. The fasting blood glucose (FBG) and weight of rats in each group were measured at 0, 1, 2, 3, and 4 weeks. Their biochemical indicators, expression of inflammatory, and antioxidant factors were measured using an automatic biochemical analyzer together with ELISA. Hematoxylin–eosin staining and periodic acid-schiff staining were used to observe the morphological changes in the kidneys of rats in each group. Finally, the expressions of related molecules and PI3K/Akt/mTOR signaling pathway proteins in renal tissues and podocytes were examined by qRT-PCR and Western blot.

**Results:**

Compared with the DN group, the FBG and weight, serum creatinine, blood urea nitrogen, total cholesterol, triacylglycerol, microalbumin, and albumin/creatinine ratio were all significantly decreased in the Baicalin treatment groups in a concentration-dependent manner. The levels of inflammatory factors in kidney tissue and podocytes were decreased. In addition, the activities of lactate dehydrogenase and malondialdehyde in tissue were decreased, while the superoxide dismutase was increased. The pathological sections showed that glomerular atrophy and glomerular basement membrane thickening caused by hyperglycemia were improved in the Baicalin treatment groups. Meanwhile, baicalin inhibited the downregulation of Nephrin and Podocin expressions and upregulation of Desmin expression caused by DN, and inhibited the expressions of p-PI3K, p-Akt, and p-mTOR proteins.

**Conclusion:**

Baicalin slows down podocyte injury caused by DN by inhibiting the activity of PI3K/Akt/mTOR signaling pathway.

## 1 Introduction

Diabetic nephropathy (DN) is a disease in which diabetes mellitus (DM) induces vascular lesions, thus promoting glomerulosclerosis [1]. DN is characterized by diffuse thickening of the glomerular basement membrane (GBM), proliferation and dilatation of the mesangial matrix, and other morphological changes, inducing renal dysfunction. DN will develop into end stage renal disease if not treated in time [2]. According to the latest statistics from the International Diabetes Federation, there are currently about 425 million DM patients worldwide and the number is expected to reach 629 million by 2045, of which 30–40% of DM patients will suffer from DN [3,4]. It has been shown that the persistent hyperglycemic state in the peripheral blood of DM patients changes hemodynamics and increases oxidative stress in the body, thus causing severe injury to local renal cells such as glomerular capillary endothelial cells and podocytes [5]. Among them, podocytes, as terminally differentiated cells, are the important functional cells in the renal glomerulus. Because of the terminal differentiation, podocytes cannot regenerate once subjected to destruction [6]. Hyperglycemia destroys the GBM, resulting in podocyte hypertrophy and detachment. So podocyte content in urine was proposed to be used as a marker to determine the early stage of DN, which is more accurate than urinary albumin [7]. Some studies have pointed out that avoiding podocyte injury is the key to prevent the occurrence of DN [8].

Baicalin (5,6,7-trihydroxyflavone) is a flavonoid extracted from scutellaria baicalensis georgi [9]. It has a wide range of biological functions, including antibacterial, antiviral, and anti-tumor functions [10,11]. Cheng showed that baicalin reduced lipopolysaccharide-induced liver inflammation in chicken by suppressing TLR4-mediated NF-*κ*B signaling pathway [12]. Meanwhile, baicalin was found to improve renal function in DN patients by inhibiting excessive inflammation and oxidative stress, and thereby effectively delaying the development of DN [13]. Baicalin is also used in clinical applications. Haixia [14] found that baicalin could reduce proteinuria in patients with early DN. Yang et al. [15] also found that baicalin can improve the renal function of patients with DN and delay the progression of DN through various pathways such as anti-inflammation and antioxidation. However, at present, the effect of baicalin on podocytes in DM-induced DN tissues and its mechanism has not been reported. Therefore, this study investigated DN rats and podocytes to provide a certain theoretical and experimental basis for subsequent research on the treatment of DM-induced DN.

## 2 Materials and methods

### 2.1 Establishment and grouping of DN rat models

A total of 30 Sprague Dawley (SD) male rats aged 8 weeks and weighing 180−200 g were selected. After 1 week of adaptive culture, the rats were randomly divided into five groups (6 rats/group). Subsequently, the DN rat models were established by intraperitoneal injection of 35 mg/kg streptozotocin (STZ) after 4 weeks of high-fat and high-sugar diet. After 72 h, the rats with fasting blood glucose (FBG) higher than 13.9 mmol/L and random blood glucose higher than 16.7 mmol/L were considered as diabetic rats. After successful modeling, the diabetic rats were divided into 4 test groups: DN group, baicalin low-concentration group (Baicalin-L), baicalin medium-concentration group (Baicalin-M), and baicalin high-concentration group (Baicalin-H). In these test groups, same amount of saline, 50 mg/kg baicalin, 100 mg/kg baicalin, and 200 mg/kg baicalin were administered, respectively, once daily for 4 weeks by gavage [16]. Meanwhile, the rats with a normal diet were intraperitoneally injected with the same amount of sodium citrate buffer for 4 weeks as the control group. After 4 weeks, 24 h urine and peripheral blood were collected, followed by anesthesia and cervical dislocation to sacrifice the rats and then their renal tissues were collected.

**Ethical approval:** This trial was approved by the ethics committee of Shenzhen Fuyong People’s Hospital.

### 2.2 Cell culture and grouping

SD male rats aged 8 weeks were injected intraperitoneally with 10% chloral hydrate, and the rats were anesthetized and then sacrificed by cervical dislocation. Their kidneys were collected under aseptic conditions, soaked and rinsed with pre-cooled sterile PBS solution. After the renal capsule was removed, the kidneys were cut into small tissue masses (1 mm^3^) using ophthalmic scissors and then collected in centrifuge tubes to digest at 150 rpm with 0.1% of collagenase type IV at 37°C. RPMI-1640 complete culture medium was added 25 min later to terminate digestion. Subsequently, the cell suspension passed through the 100, 150, and 200 mesh sieves in turn. Next the cell suspension was collected into centrifuge tube, centrifuged at 1,000 rpm at 4°C for 5 min, resuspended with complete medium, and inoculated in culture flasks to culture in an incubator (Thermo, USA) with 5% of CO_2_ at 37°C. Finally, *in vitro* podocytes of the rats were obtained. The podocytes as the Test groups were cultured with RPMI-1640 complete medium containing 30 mmol/L of glucose and then treated with 10% of serum from the rats in each test group, named as high glucose (HG) group, Baicalin-L group, Baicalin-M group, and Baicalin-H group. Meanwhile, the other part of the podocytes cultured in RPMI-1640 medium were treated with 10% of serum from the rats in the control group, named as the Control group.

### 2.3 Measurement of FBG and weight

FBG was measured in the control, DN, Baicalin-L, Baicalin-M, and Baicalin-H groups at 0, 1, 2, 3, and 4 weeks, respectively. Then 5 μL of blood collected from the caudal vein was dropped onto glucose test strips and FBG was measured by a blood glucose meter (Accu-Check Active, Roche). Their weights were also measured and recorded.

### 2.4 Detection of urine biochemical indicators

The rats in each group were fed in the promethion cages (Sable Systems International, USA), and 24 h urine was collected 4 weeks later. After the total amount of urine was determined, 3 mL of urine was centrifuged at 3,000 rpm for 10 min at 4°C, and the supernatant was taken. The 24 h microalbumin (mALB) in urine was detected using an automatic biochemical analyzer (Olympus, Japan) to calculate the urinary albumin/creatinine ratio (ACR).

### 2.5 Detection of blood biochemical indicators and inflammatory factors

The peripheral blood was collected from the orbital venous plexus of the rats and centrifuged at 3,000 rpm for 20 min. Then, the serum from the upper layer was collected and placed into new centrifuge tubes. The serum creatinine (SCr), blood urea nitrogen (BUN), total cholesterol (TC), and triacylglycerol (TG) in the serum of the rats were measured using the automatic biochemical analyzer.

In the meanwhile, the serum or podocyte supernatant of rats from each group was detected by the expressions of TNF-α, IL-1β, and IL-6 using ELISA kits (Nanjing Jiancheng Bioengineering Institute, Nanjing, China), which were operated in strict accordance with the experimental instructions.

### 2.6 Detection of oxidative damage index

Fifty milligram of kidney tissues from rats in each group were fully ground and centrifuged at 9,000 rpm for 10 min at 4°C. The supernatant was isolated to detect the activities of lactate dehydrogenase (LDH), superoxide dismutase (SOD), and malondialdehyde (MDA) in kidney tissues of rats from each group using LDH, SOD, and MDA assay kits (Nanjing Jiancheng Bioengineering Institute) according to the manufacturer’s instructions.

### 2.7 Hematoxylin–eosin (HE) staining and periodic acid-schiff (PAS) staining

The collected renal tissues were rinsed with PBS buffer. After the renal capsule was removed, the right renal tissues were fixed with 10% of neutral formaldehyde and embedded in paraffin for sectioning. The sections were stained with hematoxylin at room temperature for 5 min, followed by differentiation with hydrochloric acid alcohol. The sections appearing as blue color was washed using 1% (volume/volume%) of ammonia and then were washed in running tap water before being stained with eosin for 30 s. Finally, the sections were dehydrated with alcohol, cleared in xylene, and covered with slips. The pathological changes in the kidneys were observed under a biomicroscope.

The prepared renal tissue sections were deparaffinized at 65°C, washed with distilled water, and oxidized in 1% of periodic acid solution for 5–10 min. The sections were rinsed with distilled water again and stained with Schiff reagent for 10 min. Subsequently, the tissue sections were rinsed with sodium bisulfite solution for 3 times (2 min/time) and incubated with hematoxylin solution for 3 min. Finally, the sections were dehydrated with alcohol, treated with xylene, and covered with slips. The pathological changes in the kidneys were observed under the biomicroscope. Semiquantitative measurement of the changes was performed using Image J software according to the previous study [17].

### 2.8 MTT assay

After treatment, podocytes in the logarithmic growth phase were seeded in 96-well plates at a density of 500 cells/well and cultured for 24, 48, and 72 h, respectively. Twenty microliter of 5 mg/mL of MTT solution was added to each group of cells and continued culturing in the incubator for another 4 h. The culture supernatant in the wells was pipetted and discarded. Afterwards, 150 μL of DMSO was added and shaken for 15 min. The absorbance of each well was measured at a wavelength of 490 nm by using the enzyme mark instrument.

### 2.9 Flow cytometry (FCM)

The cells from each group were digested into a centrifuge tube by utilizing trypsin. After that, they were rinsed twice with pre-cooled sterile PBS and then adjusted the cell concentration to 5 × 10^5^ cells/mL. Two hundred microliter of cell suspension was extracted in order to add 10 μL of Annexin V-FITC followed by 10 μL of PI solution at the concentration of 20 mg/L. Finally, it was incubated in the dark at room temperature for 10 min. After adding 500 μL of PBS, apoptosis was detected by FCM.

### 2.10 qRT-PCR

Total RNA was extracted from renal tissues or cells using Trizol (Invitrogen) and the RNA concentration was measured by spectrophotometer. The total RNA was reverse transcribed into cDNA using MLV reverse transcriptase. qPCR was performed in the detection system of SYBR Green Master Mix (Promega, USA). GAPDH was used as an internal reference and the results were calculated using the 2^−△△Ct^ method. The primers used are shown in Table 1.

**Table 1:** Primer sequences

RNA	Sequences(5′ to 3′)
Nephrin	F: 5′-GCATAGCCAGAGGTGGAAATCC
	R: 5′-GAACGGTCATCACCAGCACACT
Podocin	F: 5′-GTGGAAGCTGAGGCACAAAGAC
	R: 5′-CAGCGACTGAAGAGTGTGCAAG
Desmin	F: 5′-GCGGCTAAGAACATCTCTGAGG
	R: 5′-ATCTCGCAGGTGTAGGACTGGA
GAPDH	F: 5′-CATCACTGCCACCCAGAAGACTG
	R: 5′-ATGCCAGTGAGCTTCCCGTTCAG

### 2.11 Western blot

RIPA lysis buffer was added to 50 mg of renal tissues and homogenized and then centrifuged at 12,000 rpm for 30 min at 4°C; the supernatant was taken as total protein. Meanwhile, RIPA lysis buffer was added to the podocytes and then centrifuged at 12,000 rpm for 30 min at 4°C; the supernatant was taken as total protein. The protein concentrations were determined using the BCA protein quantification kit (Thermo Fisher). In immunoblotting, 20 µg of proteins were separated using 10% of SDS-PAGE gels and transferred to polyvinylidene fluoride (PVDF) membranes. Following the blocking step with 5% of skim milk for 1 h, the membranes were incubated overnight at 4°C with the following primary antibodies: p-PI3K (Abcam UK), PI3K (Abcam UK), p-Akt (Abcam UK), Akt (Abcam UK), p-mTOR (Abcam UK), and mTOR (Abcam UK). Subsequently, they were incubated with the corresponding secondary antibodies for 1 h at room temperature. GAPDH was used as an internal reference when scanning immunoblot bands at gray scale. The gray values of the bands were analyzed using Image Lab^TM^ Software.

### 2.12 Statistical analysis

SPSS 24.0 was used for one-way analysis of variance (ANOVA) and independent sample *t*-test. The results were expressed as mean values ± standard deviation, and *p* < 0.05 indicated significant differences.

## 3 Results

### 3.1 Effects of baicalin on FBG and weight in DN rats

**Figure 1:**
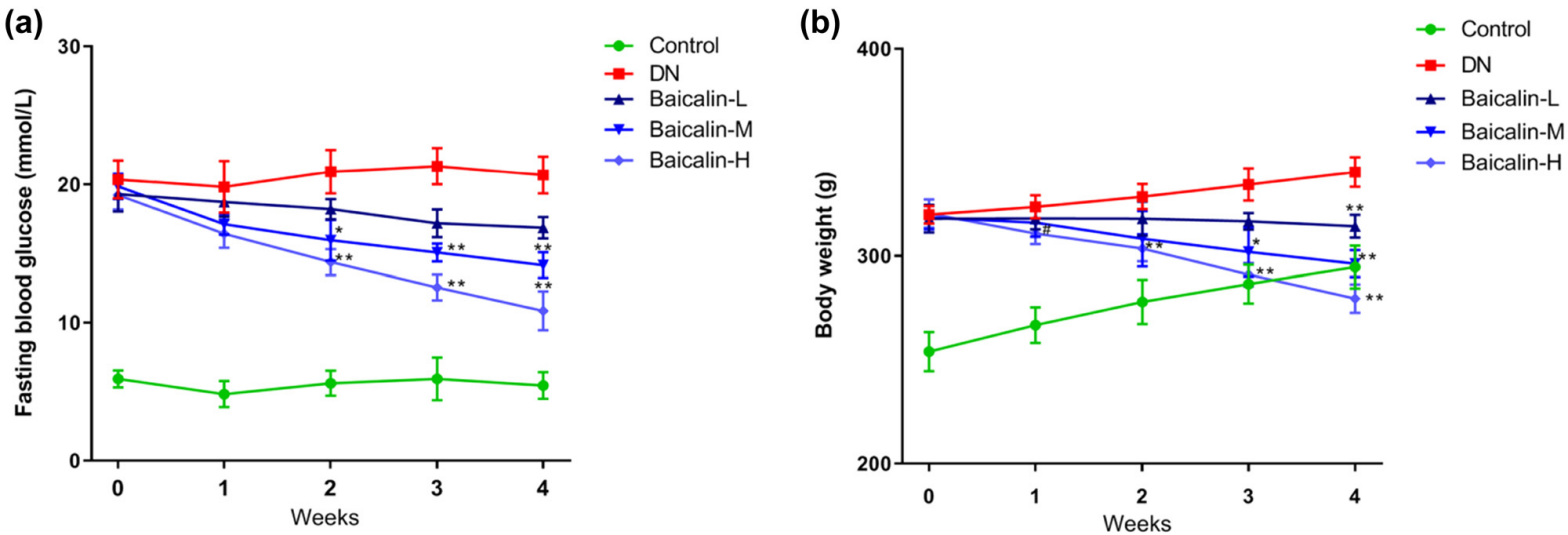
Effects of baicalin on FBG and weight in DN rats. The DN rats were administered with 50 mg/kg (Baicalin-L), 100 mg/kg (Baicalin-M), and 200 mg/kg (Baicalin-H) baicalin for 4 weeks by gavage. The FBG (a) and weight (b) of the rats in each group are measured weekly. ^#^ *p* < 0.05 vs control group; *p < 0.05 and **p < 0.01 vs DN group.

The blood glucose of rats in each group was measured by the blood glucose meter. The results showed that the level of FBG in the control group was in the normal range, while that in the DN group and Baicalin treatment groups were always at high levels. In the DN group, FBG level was higher than 16.7 mmol/L all the time. In the Baicalin treatment groups, FBG level showed a downward trend with the increase in baicalin treatment time. At the 4th week, compared with the DN group, the FBG of rats was significantly decreased in the Baicalin treatment groups (Figure 1a, *p* < 0.05), and in a concentration-dependent manner. The FBG in the Baicalin-H group was closest to that in the control group. At the 0th week, the weight of rats in the test groups was significantly higher than that in the control group. Compared with the DN group, the weights in the Baicalin treatment groups were gradually decreased (Figure 1b). These results indicated that baicalin has an inhibitory effect on blood glucose and weight in DN rats.

### 3.2 Effects of baicalin on serum and urine biochemical indicators in DN rats

**Figure 2:**
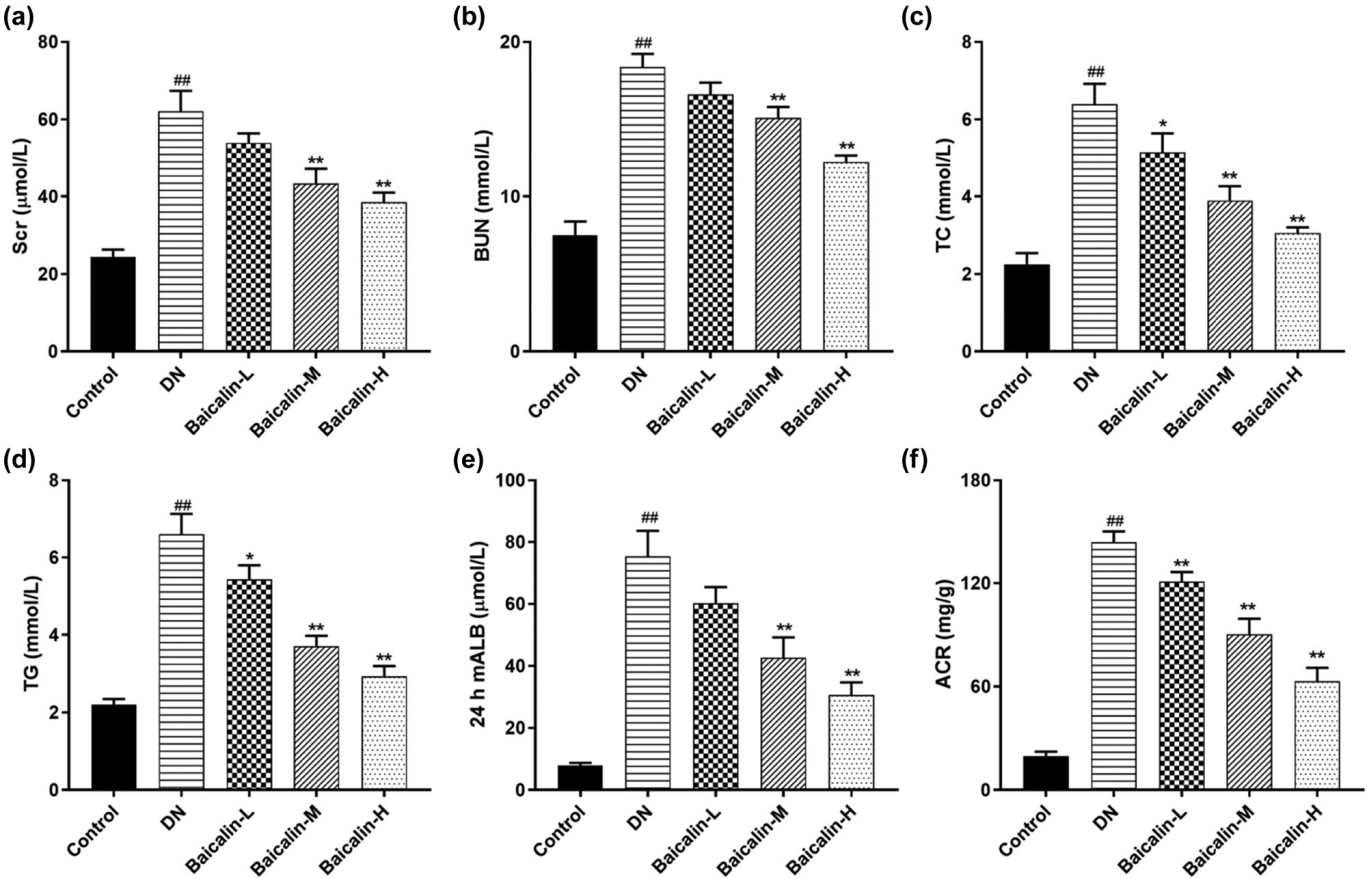
Effects of baicalin on serum and urine biochemical indicators in DN rats. The DN rats are administered with 50 mg/kg (Baicalin-L), 100 mg/kg (Baicalin-M), and 200 mg/kg (Baicalin-H) baicalin for 4 weeks by gavage. (a) SCr levels in the serum of rats in each group; (b) BUN levels in the serum of rats in each group; (c) TC levels in the serum of rats in each group; (d) TG levels in the serum of rats in each group; (e) mALB levels in the urine of rats in each group; (f) ACR levels in the urine of rats in each group. ^##^ *p* < 0.01 vs control group; *p < 0.05 and **p < 0.01 vs DN group.

The changes in serum and urine biochemical indicator in DN rats before and after baicalin treatment were observed. The results of the automatic biochemical analyzer revealed that compared with the control group, the levels of SCr, BUN, TC, and TG in the serum in the DN group were significantly increased (Figure 2a–d, *p* < 0.05), while the 24 h mALB and ACR in the urine were significantly increased (Figure 2e and f, *p* < 0.05). Compared with the DN group, the levels of SCr, BUN, TC, and TG in the Baicalin treatment groups were significantly decreased (Figure 2a–d, *p* < 0.05) in a concentration-dependent manner; the higher the baicalin concentration, the lower the levels of SCr, BUN, TC, and TG. Meanwhile, compared with the DN group, the 24 h mALB and ACR in urine in the Baicalin treatment groups were significantly decreased (Figure 2e–f, *p* < 0.05), also in a concentration-dependent manner.

### 3.3 Effects of baicalin on serum inflammatory factors and renal oxidative stress factors in DN rats

**Figure 3:**
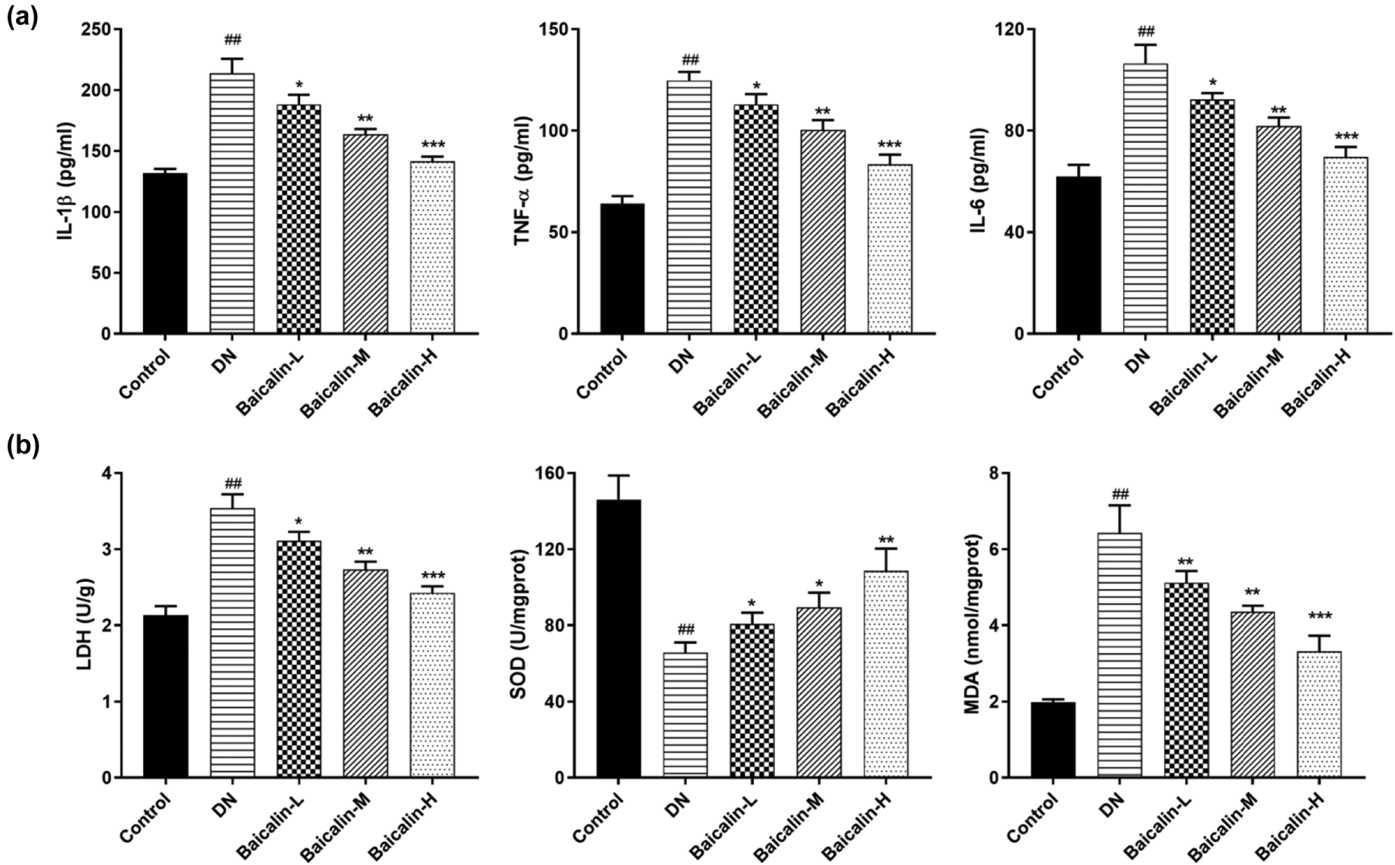
Effects of baicalin on serum inflammatory factors and renal oxidative stress factors in DN rats. The DN rats are administered with 50 mg/kg (Baicalin-L), 100 mg/kg (Baicalin-M), and 200 mg/kg (Baicalin-H) baicalin for 4 weeks by gavage. (a) the expression of inflammatory factors TNF-α, IL-1β, and IL-6 in the serum of rats in each group; (b) the activities of LDH, SOD, and MDA in the renal tissue of rats in each group; ^##^ *p* < 0.01 vs Control group; *p < 0.05, **p < 0.01, and ***p < 0.001 vs DN group.

The ELISA results showed (Figure 3a) that the expressions of inflammatory factors TNF-α, IL-1β, and IL-6 in the serum of rats from the DN group were significantly increased compared with that from the control group. Fortunately, baicalin could inhibit the expressions of TNF-α, IL-1β, and IL-6 in the serum of DN rats. Furthermore, the lower the concentration of baicalin, the lower the expressions of TNF-α, IL-1β, and IL-6.

Oxidative stress is closely related to DN incidence [18]. Figure 3b indicated that compared with the control group, the activities of LDH and MDA in the renal tissue from the DN group were significantly increased, while the activity of SOD was significantly decreased. Adding to that, compared with the DN group, the activities of LDH and MDA from different concentrations of baicalin group were markedly decreased, while the activities of SOD were significantly increased, and all of which were baicalin concentration-dependent.

### 3.4 Effects of baicalin on renal pathomorphology in DN Rats

**Figure 4:**
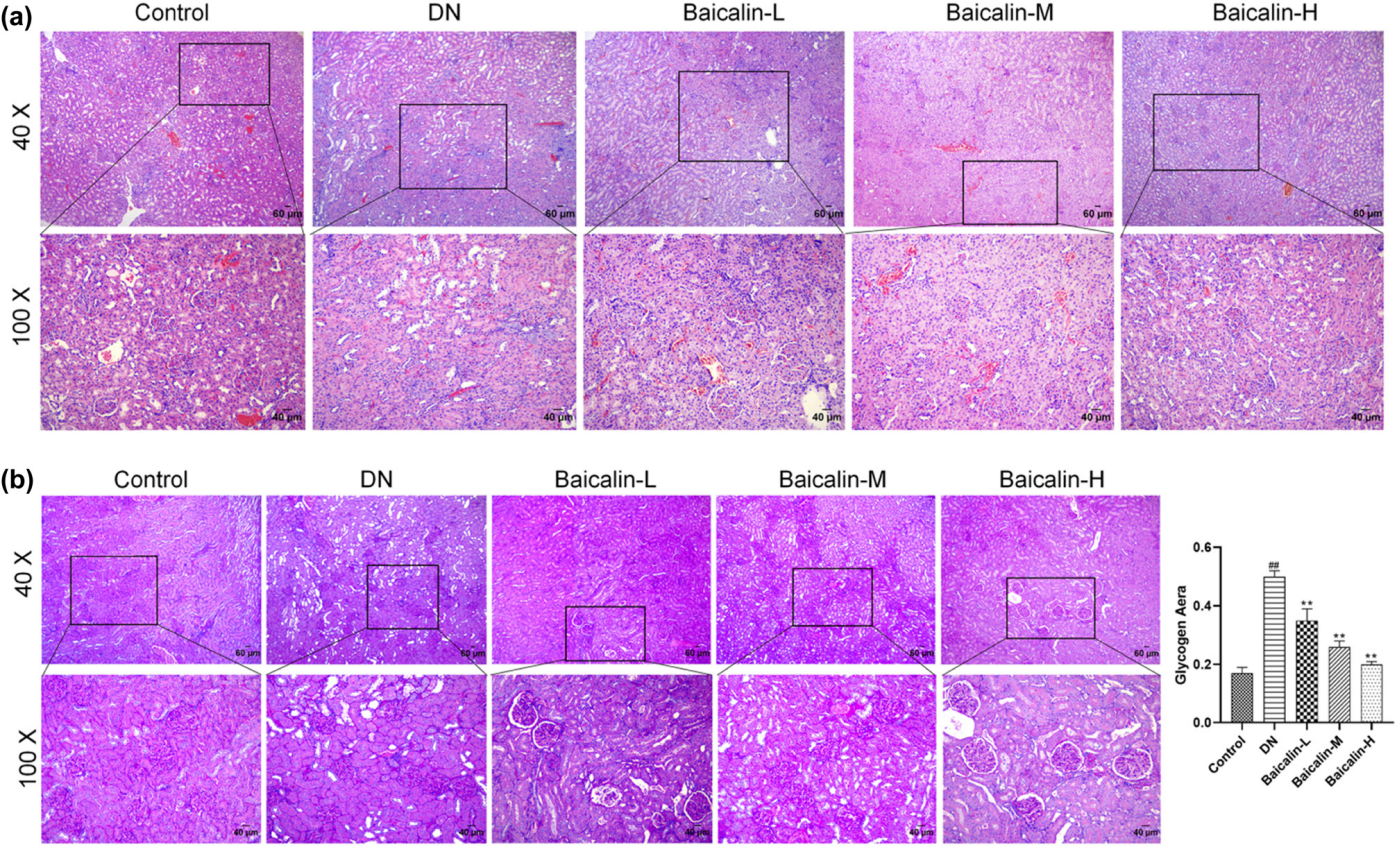
Effects of baicalin on renal pathological morphology in DN rats. The DN rats are administered with 50 mg/kg (Baicalin-L), 100 mg/kg (Baicalin-M), and 200 mg/kg (Baicalin-H) baicalin for 4 weeks by gavage, and their renal tissues are collected. (a) HE staining images of renal tissues of rats in each group; (b) PAS staining images of renal tissues of rats in each group. ^##^ *p* < 0.01 vs Control group; **p < 0.01 vs DN group.

The pathological morphological changes in the renal tissues after baicalin treatment in DN rats were examined using histopathology. The results of HE staining showed no obvious pathological changes in the renal tissues in the control group; the glomerular structure was intact, the renal tubules were arranged neatly and uniform in size, and there was no inflammatory cell infiltration. Compared with the control group, the renal tissues in the DN group were significantly damaged; the glomeruli were atrophic, the glomerular mesangial matrix was increased, the renal tubules showed vacuolar degeneration, and a large number of inflammatory cell infiltration were observed. After baicalin treatment, renal pathological injury was improved in different degrees, and the higher the baicalin concentration, the more significant the improvement (Figure 4a). The results of PAS staining showed no significant pathological changes in the renal tissues in the control group; the basement membrane was intact, and the glomerular vascular loops were thin and clear. Compared with the control group, the basement membrane in the DN group was significantly thickened. After baicalin treatment, the thickening of GBM was improved, and the higher the baicalin concentration, the more significant the improvement (Figure 4b). These results confirmed that baicalin improved renal tissue injury in DN rats in a concentration-dependent manner.

### 3.5 Effects of baicalin on the expressions of podocyte-related molecules and PI3K/Akt/mTOR signaling pathway in the renal tissues of rats

**Figure 5:**
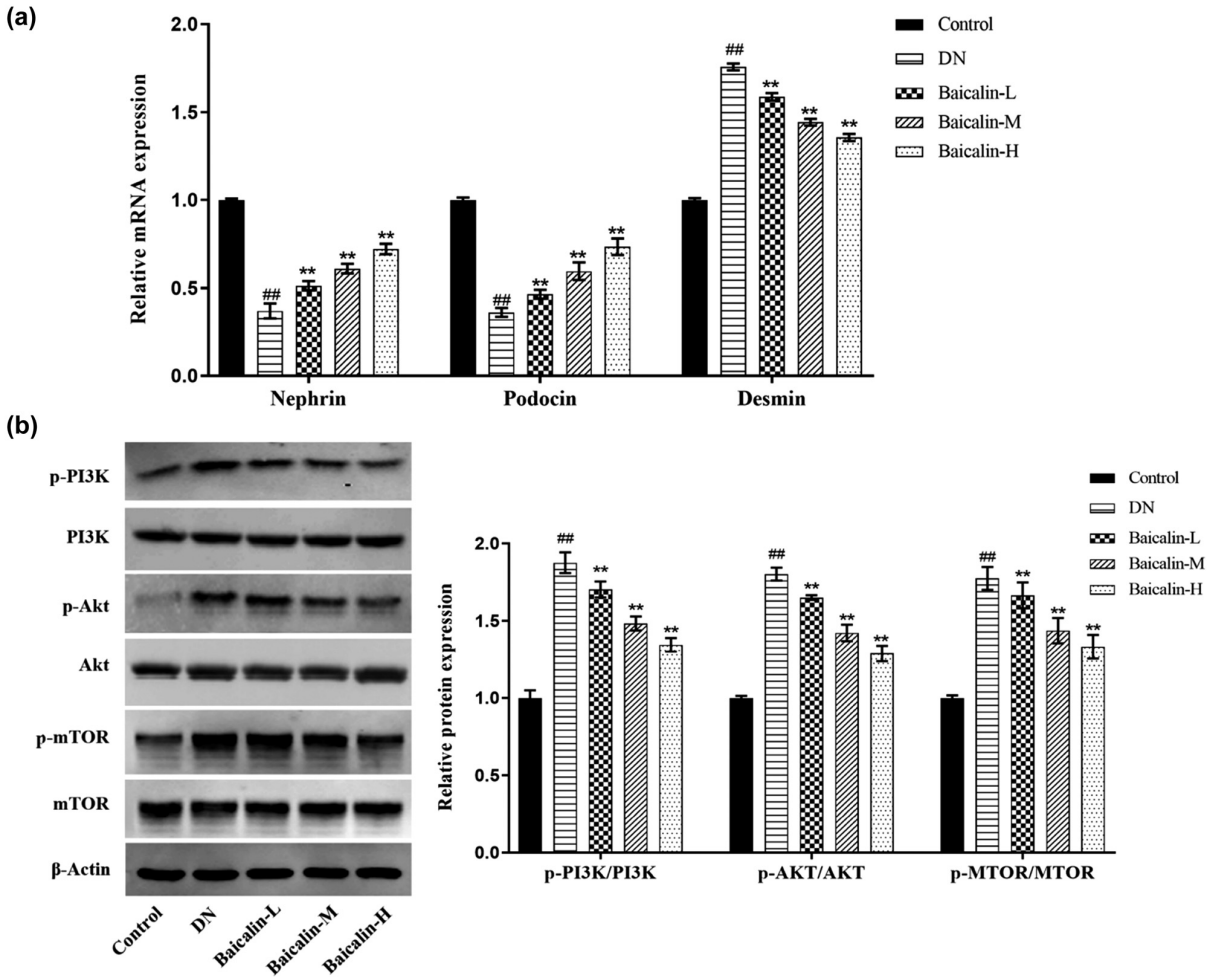
Effects of baicalin on the expressions of podocyte-related molecules and PI3K/Akt/mTOR signaling pathway in renal tissues of rats. The DN rats are administered with 50 mg/kg (Baicalin-L), 100 mg/kg (Baicalin-M), and 200 mg/kg (Baicalin-H) baicalin for 4 weeks by gavage, and their renal tissues are collected. (a) qRT-PCR to detect the mRNA expressions of Nephrin, Podocin, and Desmin in the renal tissues of rats in each group; (b) Western blot to detect the expressions of p-PI3K, PI3K, p-Akt, Akt, p-mTOR, and mTOR proteins in the renal tissues of rats in each group.^##^ *p* < 0.01 vs Control group; *p < 0.05 and **p < 0.01 vs DN group.

To investigate the molecular mechanism by which baicalin improved renal tissue injury in DN rats, the expressions of podocyte-related molecules (Nephrin, Podocin, and Desmin) and PI3K/Akt/mTOR signaling pathway proteins were detected. Compared with the control group, the mRNA expressions of Nephrin and Podocin were significantly decreased, while that of Desmin significantly increased in the renal tissues in the DN group (Figure 5a, *p* < 0.05). Compared with the DN group, the expressions of Nephrin and Podocin were significantly increased, while that of Desmin significantly decreased in the renal tissues in the baicalin treatment groups (Figure 5a, *p* < 0.05) in a concentration-dependent manner. In terms of protein, compared with the control group, the expressions of p-PI3K/PI3K, p-Akt/Akt, and p-mTOR/mTOR were significantly increased in the renal tissues in the DN group (Figure 5b, *p* < 0.05). Compared with the DN group, the expressions of p-PI3K/PI3K, p-Akt/Akt, and p-mTOR/mTOR in the baicalin treatment groups were significantly decreased (Figure 5b, *p* < 0.05) in a concentration-dependent manner.

**Figure 6:**
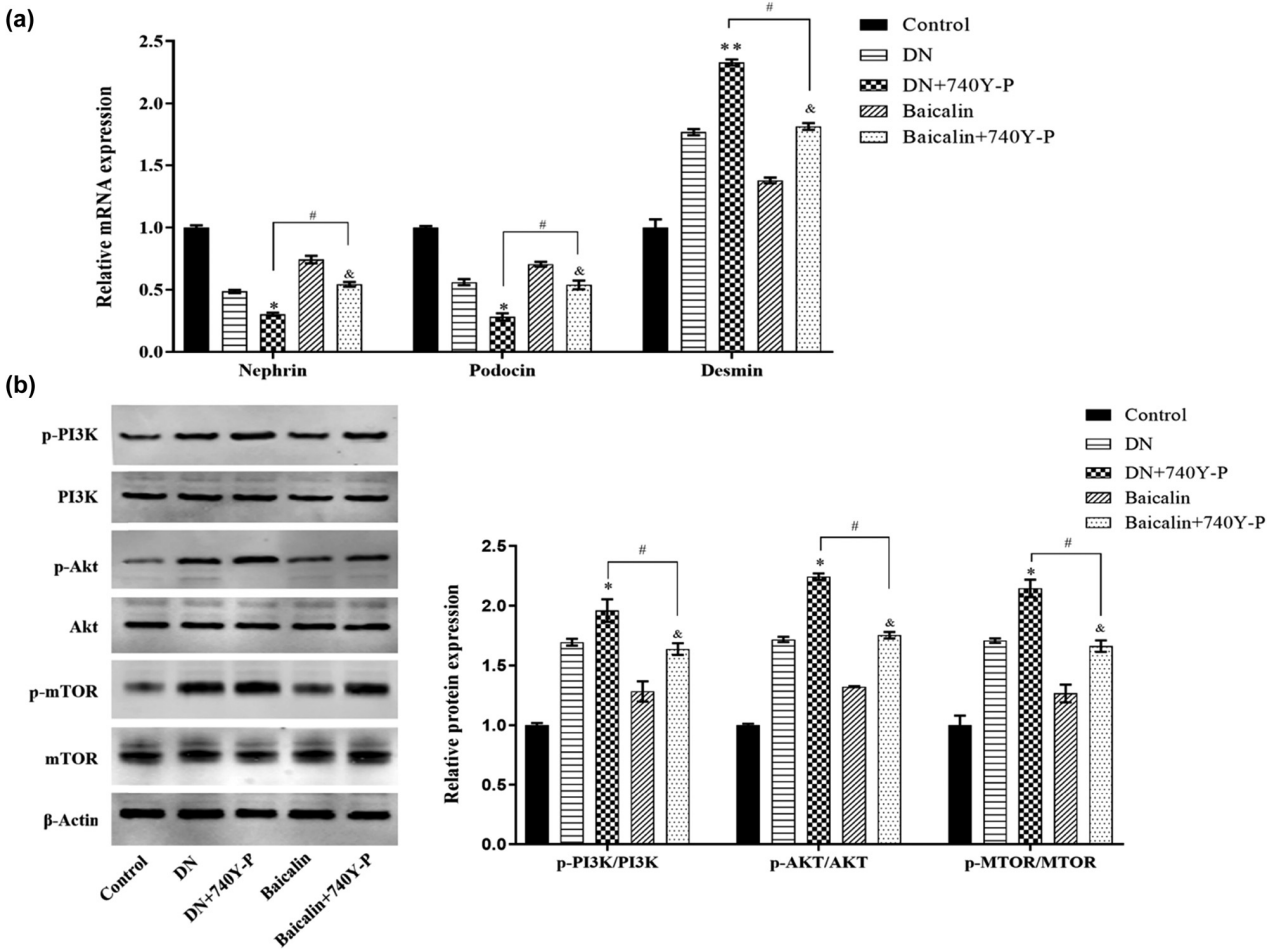
Baicalin ameliorates renal injury by inhibiting the activation of PI3K/Akt/mTOR signaling pathway in DN rats. 30 SD rats are randomly divided into 5 groups (6 rats in each group) which are control group, DN group, 740Y-P group (3.5 mg/kg PI3K activator), Baicalin group (200 mg/kg baicalin), Baicalin + 740Y-P group (200 mg/kg baicalin + 3.5 mg/kg 740Y-PA), with 4 weeks of gavage. (a) qRT-PCR to detect the mRNA expressions of Nephrin, Podocin, and Desmin in the kidney tissues of rats in each group; (b) Western blot to detect the protein expressions of p-PI3K, PI3K, p-Akt, Akt, p-mTOR, and mTOR in the kidney tissues of rats in each group. *p < 0.05 vs DN group; ^&^ *p* < 0.05 vs Baicalin group; and ^#^ *p* < 0.05 vs DN + 740Y-P group.

The results further confirmed that baicalin ameliorates renal injury in DN rats by inhibiting PI3K/Akt/mTOR. Besides, after the PI3K/Akt signaling pathway was activated via 740Y-P, the results showed that (Figure 6a and b) the expressions of Nephrin and Podocin in the renal tissue of rats from the DN group were inhibited, but the protein expression of Desmin, like p-PI3K, p-Akt, and p-mTOR, was promoted. Expressions of these proteins in rat kidney tissue were reversed upon baicalin treatment. In short, the results confirmed that baicalin could ameliorate renal injury by inhibiting the activation of PI3K/Akt/mTOR signaling pathway in DN rats.

### 3.6 Effect of baicalin on podocyte injury induced by HG

**Figure 7:**
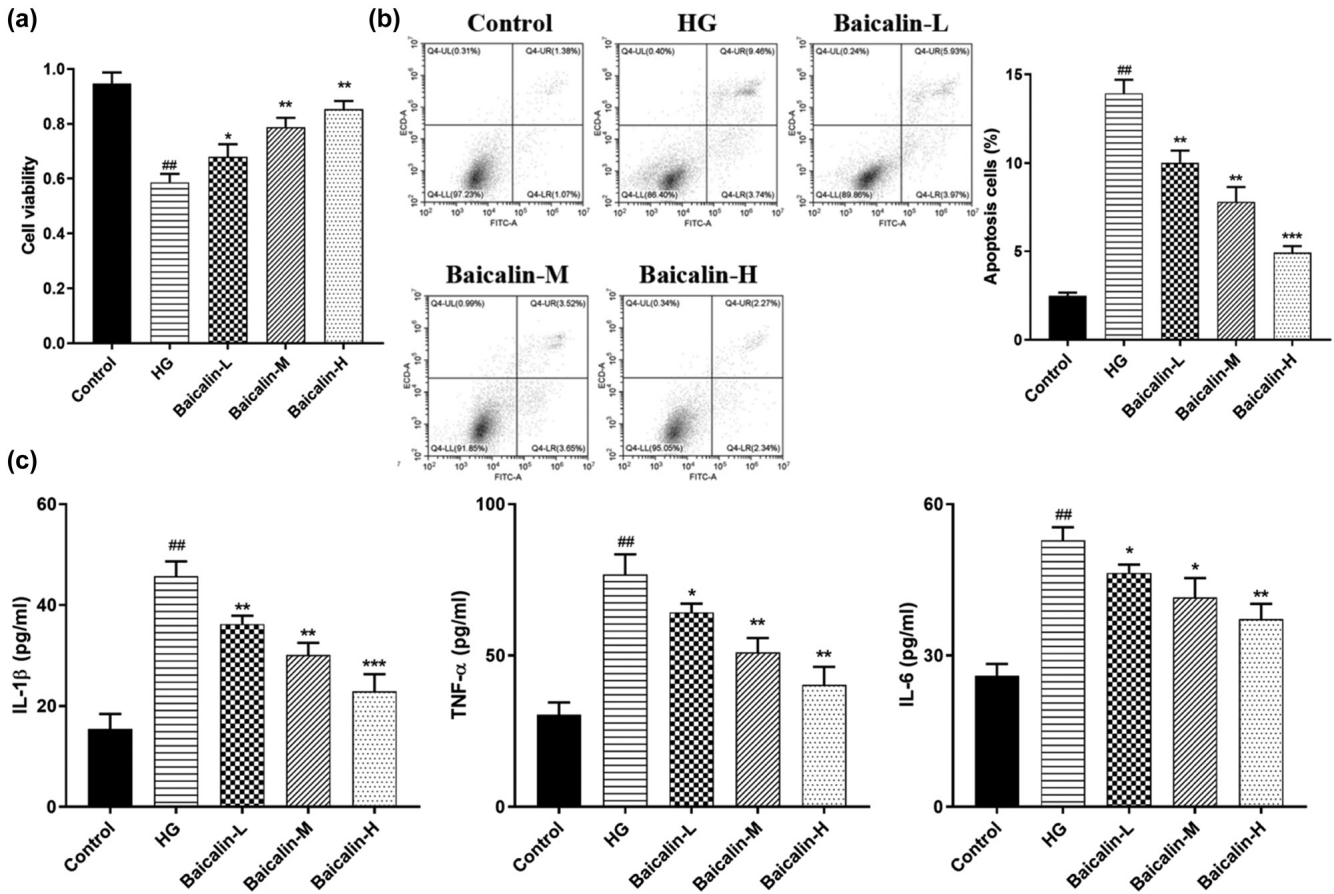
Effect of baicalin on podocyte injury induced by HG. Primary culture of rat podocytes was carried out and podocyte models of HG were induced using 30 mmol/L of glucose and then the podocytes were treated with 10% of serum from the rats in each test group. (a) MTT assay for podocyte viability; (b) flow assay for podocyte apoptosis; (c) ELISA for the expression of inflammatory factors TNF-α, IL-1β, and IL-6. ^##^ *p* < 0.01 vs control group; *p < 0.05, **p < 0.01, and ***p < 0.001 vs HG group.

In order to further confirm the results of *in vitro* trails, primary culture of rat podocytes was carried out and podocyte models of HG were induced using 30 mmol/L of glucose and then the podocytes were treated with 10% of serum from the rats in each test group. The results of Figure 7a–c show that compared with the control group, the viability of podocytes in the HG group was significantly decreased with an increase in the rate of apoptosis. However, the expressions of inflammatory factors such as TNF-α, IL-1β, and IL-6 were significantly upregulated. Baicalin, on the one hand, could promote the proliferation of podocytes. On the other hand, it could inhibit apoptosis and the expression of TNF-α, IL-1β, and IL-6.

### 3.7 Effect of baicalin on PI3K/Akt/mTOR signaling pathway after podocyte injury induced by HG

**Figure 8:**
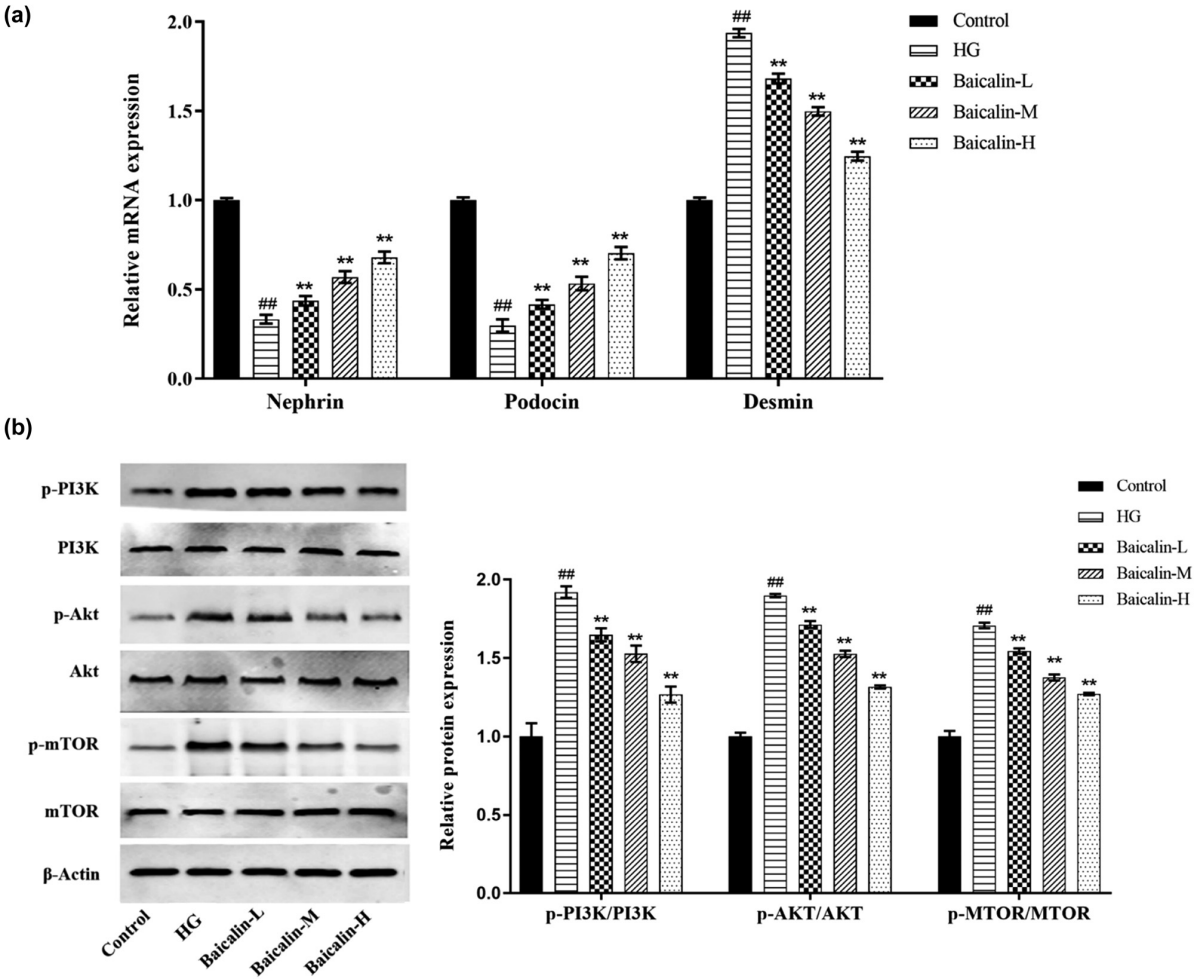
*In vitro* trails to detect effects of baicalin on rat podocytes. Primary culture of rat podocytes is carried out and the podocyte models of HG are induced using 30 mmol/L of glucose and then the podocytes are treated with 10% of serum from the rats in each test group. (a) qRT-PCR to detect the mRNA levels of Nephrin, Podocin, and Desmin in podocytes of each group; (b) Western blot to detect the expressions of p-PI3K, PI3K, p-Akt, Akt, p-mTOR, and mTOR proteins in podocytes of each group. ^##^ *p* < 0.01 vs Control group; *p < 0.05 and **p < 0.01 vs DN group.

Compared with the Control group, the mRNA expressions of Nephrin and Podocin were significantly decreased, while that of Desmin significantly increased in the HG group (Figure 8a, *p* < 0.05). Compared with the HG group, the mRNA expressions of Nephrin and Podocin were significantly upregulated, while that of Desmin was significantly downregulated in the Baicalin treatment groups (Figure 8a, *p* < 0.05) in a concentration-dependent manner. In terms of protein, compared with the control group, the expressions of p-PI3K/PI3K, p-Akt/Akt, and p-mTOR/mTOR were significantly increased in the HG group (Figure 8b, *p* < 0.05). Compared with the HG group, the expressions of p-PI3K/PI3K, p-Akt/AKT, and p-mTOR/mTOR proteins were significantly decreased in the Baicalin treatment groups (Figure 8b, *p* < 0.05) in a concentration-dependent manner. These results of trails *in vitro* and *in vivo* were similar.

## 4 Discussion

Current treatments for DN patients include diet control, exercise enhancement, weight control, and drug therapy. The first three are known as non-drug therapies, in which weight control can effectively control diabetes and slow down the development of DN [19]. In addition, drug therapy is to control blood glucose in DN patients. Many studies have shown that blood glucose controlled within the normal range is the basis for the treatment of DN [20], while poor blood glucose control induces DN development as well as other serious microvascular complications [21]. In addition, the control of urinary protein is also the key to the treatment of DN [22]. In this study, diabetic rat models with DN were established by high-fat and high-sugar diet and STZ injection. In the test groups, DN rats had significantly increased FBG and weight, significant proteinuria (upregulation of 24 h mALB and urinary ACR), increased SCr, BUN, TC, and TG in serum, and significant changes in podocyte-related molecules (downregulation of Nephrin and Podocin expressions and upregulation of Desmin expression). Many studies have pointed out that baicalin can reduce diabetic kidney injury. Zhang et al. [23] showed that baicalin significantly inhibited DM-induced fibrosis of proximal renal tubular epithelial cells. Zheng et al. [24] demonstrated that baicalin–lysin conjugate ameliorated renal fibrosis in STZ-induced DN rats. Li et al. revealed that baicalin could prevent the apoptosis of podocytes due to HG, thereby delaying the development of DN [25]. In this study, during the treatment of DN rats with baicalin, FBG and body weight of rats treated with different concentrations of baicalin showed a downward trend, and longer treatment time and higher concentration cause more significant decrease in FBG and weight in rats. Meanwhile, baicalin significantly inhibited the levels of SCr, BUN, TC, and TG in the blood and urinary mALB and ACR. Furthermore, by observing the pathological sections of the kidneys, it can be seen that baicalin improved GBM thickening and glomerular atrophy caused by hyperglycemia. Baicalin could also significantly upregulate the expressions of Nephrin and Podocin, but downregulate the expression of Desmin. Some studies have pointed out that upregulation of Nephrin and Podocin and downregulation of Desmin are beneficial to protect the morphology, structure, and function of podocytes, and to inhibit the epithelial-mesenchymal transition of the podocytes [26]. Therefore, the result indicated that baicalin maintained the homeostasis of podocyte-related functional proteins to protect the normal functions of podocytes. All these studies confirmed that baicalin could improve renal injury in DN rats.

An increasing number of studies have shown that hyperglycemia induced oxidative stress or inflammatory factors, leading to podocyte detachment from GBM and apoptosis, which is the major factor in the early development of DN [18]. In this study, we found that the expressions of inflammatory factors, namely, TNF-α, IL-1β, and IL-6 were all upregulated in the serum of rats in the DN group. Furthermore, the activities of LDH and MDA were increased, but the activity of SOD was decreased in the renal tissue. At the same time, HG-induced podocyte viability was reduced, but apoptotic ability was enhanced, along with upregulation of TNF-α, IL-1β, and IL-6 expressions. Baicalin, on the other hand, could reverse these changes. These results confirmed that baicalin could ameliorate the damage of renal podocytes in DN rats. DM will cause hemodynamic changes, leading to the abnormal activation of PI3K/Akt/mTOR pathway. This abnormal activation induces autophagy of podocytes, thus inhibiting the adhesive ability of podocytes and resulting in detachment of podocytes from GBM [27]. Many studies have explored new therapeutic ideas for DN based on the effect mention above. Wu et al. [28] found that Huangkui capsule alleviated glomerular pathological changes in the early stage of DN by inhibiting the activity of Akt/mTOR signaling pathway. Huang et al. [29] revealed that Notoginsenoside R1 could inhibit autophagy and apoptosis of podocytes to reduce glucose-induced podocyte injury through inhibiting the activity of the PI3K/Akt/mTOR signaling pathway. Wu et al. [30] also found that curcumin inhibited the autophagic transformation of podocytes through suppressing the activity of this signaling pathway, thus reducing the damage caused by DN to the body. Therefore, it is speculated that baicalin also has a benign effect on the development of DN by regulating this signaling pathway. From our results of Western blot, baicalin significantly inhibited the expression of p-PI3K/PI3K, p-Akt/Akt, p-mTOR/mTOR in both *in vivo* and *in vitro* experiments. So, we believed the possibility that baicalin protected podocytes by downregulating the activity of the PI3K/Akt/mTOR signaling pathway.

## 5 Conclusion

In summary, baicalin can not only reduce the increase in FBG, body weight, and biochemical indicator in serum and urine due to DM, but also slow down the damage of podocytes caused by hyperglycemia by inhibiting the activity of PI3K/Akt/mTOR signaling pathway, thereby delaying the progression of DM-induced DN. The results of this trial provide effective data for the clinical application of baicalin in the treatment of DN.

## References

[1] Rossing P , Astrup AS , Smidt UM , Parving HH . Monitoring kidney function in diabetic nephropathy. Diabetologia. 1994;37(7):708–12. 10.1007/BF00417696 7958543

[2] Verma AK , Chandra S , Singh RG , Singh TB , Srivastava S , Srivastava R . Serum prolidase activity and oxidative stress in diabetic nephropathy and end stage renal disease: a correlative study with glucose and creatinine. Biochem Res Int. 2014;2014:291458. 10.1155/2014/291458 PMC4172940 25276429

[3] Cho NH , Shaw JE , Karuranga S , Huang Y , Da RFJ , Ohlrogge AW , et al. IDF diabetes atlas: global estimates of diabetes prevalence for 2017 and projections for 2045. Diabetes Res Clin Pract. 2018;138:271–81. 10.1016/j.diabres.2018.02.023 29496507

[4] Cho NH . Q&A: Five questions on the 2015 IDF Diabetes Atlas. Diabetes Res Clin Pract. 2016;115:157–9. 10.1016/j.diabres.2016.04.048 27242128

[5] Najafian B , Alpers CE , Fogo AB . Pathology of human diabetic nephropathy. Contrib Nephrol. 2011;170:36–47. 10.1159/000324942 21659756

[6] Maezawa Y , Takemoto M , Yokote K . Cell biology of diabetic nephropathy: roles of endothelial cells, tubulointerstitial cells and podocytes. J Diabetes Investig. 2015;6(1):3–15. 10.1111/jdi.12255 PMC4296695 25621126

[7] Gluhovschi C , Gluhovschi G , Petrica L , Timar R , Velciov S , Ionita I , et al. Urinary biomarkers in the assessment of early diabetic nephropathy. J Diabetes Res. 2016;2016:4626125. 10.1155/2016/4626125 PMC4927990 27413755

[8] Pan Y , Jiang S , Hou Q , Qiu D , Shi J , Wang L , et al. Dissection of glomerular transcriptional profile in patients with diabetic nephropathy: SRGAP2a protects podocyte structure and function. Diabetes. 2018;67(4):717–30. 10.2337/db17-0755 29242313

[9] Luo J , Dong B , Wang K , Cai S , Liu T , Cheng X , et al. Baicalin inhibits biofilm formation, attenuates the quorum sensing-controlled virulence and enhances Pseudomonas aeruginosa clearance in a mouse peritoneal implant infection model. PLoS One. 2017;12(4):e0176883. 10.1371/journal.pone.0176883 PMC5409170 28453568

[10] Novy P , Urban J , Leuner O , Vadlejch J , Kokoska L . In vitro synergistic effects of baicalin with oxytetracycline and tetracycline against Staphylococcus aureus. J Antimicrob Chemother. 2011;66(6):1298–300. 10.1093/jac/dkr108 21421582

[11] Li HY , Hu J , Zhao S , Yuan ZY , Wan HJ , Lei F , et al. Comparative study of the effect of baicalin and its natural analogs on neurons with oxygen and glucose deprivation involving innate immune reaction of TLR2/TNFalpha. J Biomed Biotechnol. 2012;2012:267890. 10.1155/2012/267890 PMC3321472 22536016

[12] Cheng P , Wang T , Li W , Muhammad I , Wang H , Sun X , et al. Baicalin alleviates lipopolysaccharide-induced liver inflammation in chicken by suppressing TLR4-mediated NF-kappaB pathway. Front Pharmacol. 2017;8:547. 10.3389/fphar.2017.00547 PMC5563358 28868036

[13] Sun QR , Zhang X , Fang K . Phenotype of vascular smooth muscle cells (VSMCs) is regulated by miR-29b by targeting Sirtuin 1. Med Sci Monit. 2018;24:6599–607. 10.12659/MSM.910068 PMC6354642 30231015

[14] Haixia L . Effects of baicalin on renal function and oxidative stress in early diabetic nephropathy. Chin J Mod Drug Appl. 2013;7(20):142–3.

[15] Yang M , Kan L , Wu L , Zhu Y , Wang Q . Effect of baicalin on renal function in patients with diabetic nephropathy and its therapeutic mechanism. Exp Ther Med. 2019;17(3):2071–6. 10.3892/etm.2019.7181 PMC6396004 30867693

[16] Cai Y , Ma W , Xiao Y , Wu B , Li X , Liu F , et al. High doses of baicalin induces kidney injury and fibrosis through regulating TGF-beta/Smad signaling pathway. Toxicol Appl Pharmacol. 2017;333:1–9. 10.1016/j.taap.2017.08.003 28803990

[17] Chen J , Hu Y , Mou X , Wang H , Xie Z . Amygdalin alleviates renal injury by suppressing inflammation, oxidative stress and fibrosis in streptozotocin-induced diabetic rats. Life Sci. 2021;265:118835. 10.1016/j.lfs.2020.118835 33253723

[18] Welsh GI , Saleem MA . The podocyte cytoskeleton–key to a functioning glomerulus in health and disease. Nat Rev Nephrol. 2011;8(1):14–21. 10.1038/nrneph.2011.151 22025085

[19] Harrison AL , Shields N , Taylor NF , Frawley HC . Exercise improves glycaemic control in women diagnosed with gestational diabetes mellitus: a systematic review. J Physiother. 2016;62(4):188–96. 10.1016/j.jphys.2016.08.003 27637772

[20] Schorr SG , Hammes HP , Muller UA , Abholz HH , Landgraf R , Bertram B . The prevention and treatment of retinal complications in diabetes. Dtsch Arztebl Int. 2016;113(48):816–23. 10.3238/arztebl.2016.0816 PMC5241793 28073426

[21] Fioretto P , Bruseghin M , Berto I , Gallina P , Manzato E , Mussap M . Renal protection in diabetes: role of glycemic control. J Am Soc Nephrol. 2006;17(4 Suppl 2):S86–9. 10.1681/ASN.2005121343 16565255

[22] Kistler AD , Peev V , Forst AL , El HS , Altintas MM , Reiser J . Enzymatic disease of the podocyte. Pediatr Nephrol. 2010;25(6):1017–23. 10.1007/s00467-009-1425-1 PMC4109305 20130922

[23] Zhang S , Xu L , Liang R , Yang C , Wang P . Baicalin suppresses renal fibrosis through microRNA-124/TLR4/NF-kappaB axis in streptozotocin-induced diabetic nephropathy mice and high glucose-treated human proximal tubule epithelial cells. J Physiol Biochem. 2020;76(3):407–16. 10.1007/s13105-020-00747-z 32500512

[24] Zheng XP , Nie Q , Feng J , Fan XY , Jin YL , Chen G , et al. Kidney-targeted baicalin-lysozyme conjugate ameliorates renal fibrosis in rats with diabetic nephropathy induced by streptozotocin. BMC Nephrol. 2020;21(1):174. 10.1186/s12882-020-01833-6 PMC7216346 32398108

[25] Li J , Ling Y , Yin S , Yang S , Kong M , Li Z . Baicalin serves a protective role in diabetic nephropathy through preventing high glucose-induced podocyte apoptosis. Exp Ther Med. 2020;20(1):367–74. 10.3892/etm.2020.8701 PMC7296293 32550886

[26] Sun H , Wang W , Han P , Shao M , Song G , Du H , et al. Astragaloside IV ameliorates renal injury in db/db mice. Sci Rep. 2016;6:32545. 10.1038/srep32545 PMC5009300 27585918

[27] Takeuchi H , Kondo Y , Fujiwara K , Kanzawa T , Aoki H , Mills GB , et al. Synergistic augmentation of rapamycin-induced autophagy in malignant glioma cells by phosphatidylinositol 3-kinase/protein kinase B inhibitors. Cancer Res. 2005;65(8):3336–46. 10.1158/0008-5472.CAN-04-3640 15833867

[28] Wu W , Hu W , Han WB , Liu YL , Tu Y , Yang HM , et al. Inhibition of Akt/mTOR/p70S6K signaling activity with huangkui capsule alleviates the early glomerular pathological changes in diabetic nephropathy. Front Pharmacol. 2018;9:443. 10.3389/fphar.2018.00443 PMC5976825 29881349

[29] Huang G , Zou B , Lv J , Li T , Huai G , Xiang S , et al. Notoginsenoside R1 attenuates glucose-induced podocyte injury via the inhibition of apoptosis and the activation of autophagy through the PI3K/Akt/mTOR signaling pathway. Int J Mol Med. 2017;39(3):559–68. 10.3892/ijmm.2017.2864 PMC5360354 28112381

[30] Wu W , Geng H , Liu Z , Li H , Zhu Z . Effect of curcumin on rats/mice with diabetic nephropathy: a systematic review and meta-analysis of randomized controlled trials. J Tradit Chin Med. 2014;34(4):419–29. 10.1016/s0254-6272(15)30041-8 25185359

